# Hospitalized Children with 2009 Pandemic Influenza A (H1N1): Comparison to Seasonal Influenza and Risk Factors for Admission to the ICU

**DOI:** 10.1371/journal.pone.0015173

**Published:** 2010-12-15

**Authors:** Dayanand Bagdure, Donna J. Curtis, Emily Dobyns, Mary P. Glodé, Samuel R. Dominguez

**Affiliations:** 1 Department of Pediatric Critical Care, The Children's Hospital, University of Colorado School of Medicine, Aurora, Colorado, United States of America; 2 Department of Pediatric Infectious Diseases, The Children's Hospital, University of Colorado School of Medicine, Aurora, Colorado, United States of America; Duke-NUS Graduate Medical School, Singapore

## Abstract

**Background:**

Limited data are available describing the clinical presentation and risk factors for admission to the intensive care unit for children with 2009 H1N1 infection.

**Methods:**

We conducted a retrospective chart review of all hospitalized children with 2009 influenza A (H1N1) and 2008–09 seasonal influenza at The Children's Hospital, Denver, Colorado.

**Results:**

Of the 307 children identified with 2009 H1N1 infections, the median age was 6 years, 61% were male, and 66% had underlying medical conditions. Eighty children (26%) were admitted to the ICU. Thirty-two (40%) of the ICU patients required intubation and 17 (53%) of the intubated patients developed acute respiratory distress syndrome (ARDS). Four patients required extracorporeal membrane oxygenation. Eight (3%) of the hospitalized children died. Admission to the ICU was significantly associated with older age and underlying neurological condition. Compared to the 90 children admitted during the 2008–09 season, children admitted with 2009 H1N1 influenza were significantly older, had a shorter length of hospitalization, more use of antivirals, and a higher incidence of ARDS.

**Conclusions:**

Compared to the 2008–09 season, hospitalized children with 2009 H1N1 influenza were much older and had more severe respiratory disease. Among children hospitalized with 2009 H1N1 influenza, risk factors for admission to the ICU included older age and having an underlying neurological condition. Children under the age of 2 hospitalized with 2009 H1N1 influenza were significantly less likely to require ICU care compared to older hospitalized children.

## Introduction

In June 2009, the World Health Organization declared that the spread of the 2009 influenza A (H1N1) virus had reached pandemic levels [1]. Certainly the numbers affected and the media coverage were different for 2009 H1N1 than for a typical influenza season, but whether 2009 H1N1 was different clinically from seasonal influenza is unclear.

In this study, we present our experience with children admitted to a tertiary care children's hospital with 2009 H1N1 infection and provide a comparison to the prior influenza season in order to provide a context for our findings. Our primary goals were to describe the demographic characteristics, clinical features, laboratory findings, and outcomes of consecutively, hospitalized children with H1N1 infection and to compare children admitted to the Intensive Care Unit (ICU) with those admitted to the pediatric floor. Our secondary goal was to compare children hospitalized with 2009 H1N1 to those admitted with 2008–09 seasonal influenza.

## Methods

### Patients and setting

We conducted a retrospective chart review of hospitalized patients at The Children's Hospital (TCH), Denver, Colorado, an academic, tertiary-care, 314-bed hospital serving Colorado and the surrounding states. TCH's primary catchment population is the Denver metro area, which has a population of approximately 2.5 million people. The hospital has approximately 13,000 inpatient admissions and 117,000 emergency room/urgent care visits a year.

For the 2009 H1N1 cohort, eligible patients included all children who were admitted to the pediatric floor or to the intensive care unit who had a respiratory specimen positive for the 2009 influenza A H1N1 virus from May 1, 2009 to November 30, 2009. For seasonal influenza patients, all patients admitted with a positive respiratory specimen for seasonal influenza A or B from December 2008 to May 2009 were included in the cohort. A list of patients was generated by identifying respiratory samples which were positive for influenza by immunoassay (IA), direct fluorescent antibody (DFA) or real-time reverse transcriptase-polymerase-chain-reaction (RT-PCR). Samples that were positive by IA or DFA, but negative by RT-PCR were excluded. Respiratory specimens consisted primarily of nasopharyngeal washes.

IA was performed using BinaxNOW® Influenza A and B kits (Binax, Inc. Scarborough, Maine,USA). Respiratory specimens were extracted using viral RNA kits on a BioRobot EZ1 extractor (Qiagen, Valencia, CA) per manufacturer instructions and tested in the clinical laboratory by multipex PCR for 12 respiratory viruses and subtypes by xTag ® Respiratory Virus Panel, RVP (Luminex Molecular Diagnostics, Toronto CN). Previously published reports[2], in addition to our clinical laboratory's experience (unpublished data), have shown that specimens identified as nontypeable influenza A using this assay were uniformly confirmed to be the 2009 pandemic influenza A (H1N1) strain using the published CDC assay. Based on these results, specimens were considered to be 2009 H1N1 positive if determined to be influenza A and non typeable by PCR.

Clinical, laboratory, and demographic information was obtained by chart review by physicians in the Departments of Critical Care and Infectious Diseases. The day of admission was considered hospital day 0. Obesity was defined as body mass index above the 95^th^ percentile for age and sex [3]. Bacterial infections were defined as any positive culture from a normally sterile site. A diagnosis of asthma was based on patient report of medical history. Hypoxia was defined by use of oxygen during the hospitalization. Acute respiratory distress syndrome was defined as acute onset, non cardiogenic edema with bilateral pulmonary infiltrates on chest radiograph and a ratio of partial pressure of oxygen in arterial blood (PaO_2_) to fraction concentration of oxygen in inspired air (FiO_2_) of less than or equal to 200 or requiring high peak end expiratory pressure to maintain inspired oxygen levels at non toxic levels [4]. The criteria for admission to the hospital and the ICU during the 2008–09 and 2009 H1N1 season remained the same.

The protocol and standardized data collection form were approved by the Colorado Multiple Institutional Review Board. Waiver of informed consent was approved for retrospective chart review of study participants. Study data were collected and managed using REDCap electronic data capture tools hosted at The University of Colorado [5].

### Statistical analysis

We evaluated the characteristics of the children who had 2009 H1N1 influenza admitted to the pediatric floor and the ICU and compared them to identify potential risk factors for more severe disease. We also compared children hospitalized with 2009 H1N1 to those who were admitted with 2008–09 seasonal influenza to identify potentially unique clinical characteristics of 2009 H1N1. For categorical variables, the percentages of patients in each category were calculated. Wilcoxon rank-sum test was used for continuous variables and Fisher's exact test or the chi-square test for dichotomous variables. Percentages of expected characteristics of all hospitalized patients based on published population rates [6] were compared to observed characteristics using the chi square statistic. Factors that had a significant association (p<0.05) with ICU admission were put into a full model for multiple logistic regression analyses. A backward selection method was performed for model fitting and predictors with the highest p-value were taken out from the model one at a time until achievement of the most parsimonious model. Both p-value of individual effect and Akaike Information Criterion were used to assess model fitting. All analyses for this study were performed with SAS software, version 9.2.

## Results

### 2009 H1N1 Demographics

**Figure 1:**
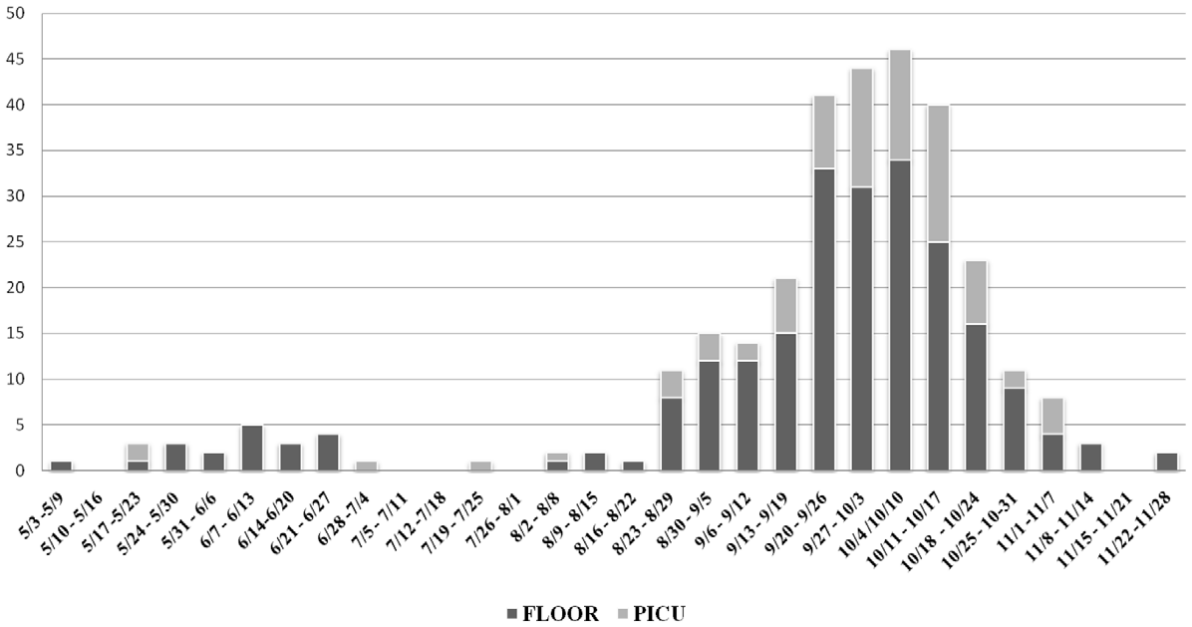
Children hospitalized with 2009 H1N1 influenza infection by week of admission.

**Figure 2:**
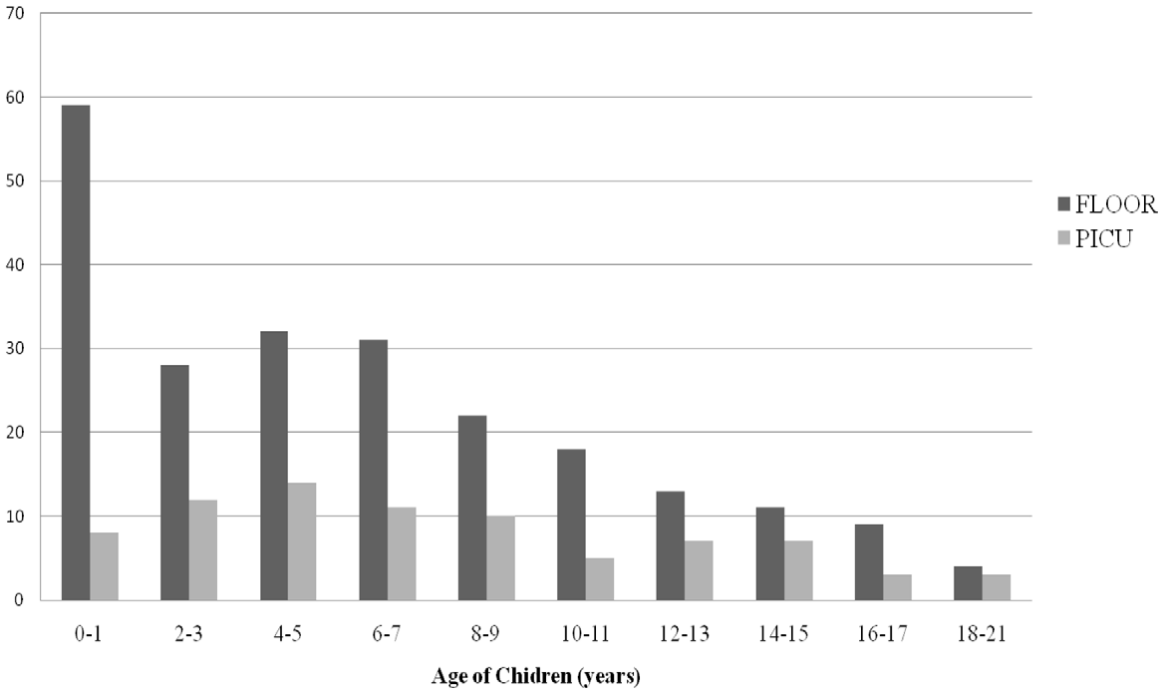
Number of children hospitalized with 2009 H1N1 influenza infection by age group.

From May 1 to November 30, 2009, 307 children with 2009 H1N1 influenza infection were hospitalized of which 80 (26%) were admitted to the ICU (Figure 1). The demographics of children admitted with 2009 H1N1 influenza are shown in Table 1. The median age of ICU patients was 6.7 years and of floor patients was 5.7 years (p = 0.02). The age grouping with the largest number of children admitted to the ICU was 4–5 years of age, whereas the largest age group of children admitted to the floor was less than 2 years of age (Figure 2).

**Table 1:** Demographics and underlying medical conditions in children hospitalized with 2009 H1N1 influenza infection.

	TOTAL	FLOOR	PICU	p value#
	N = 307	N = 227 (74%)	N = 80 (26%)	
DEMOGRAPHICS	Number (%)	Number (%)	Number (%)	
Male sex	186 (61)+	132 (58)	54 (68)	0.15
Hispanic/Latino	117 (38)	88 (39)	29 (36)	
American Indian	4 (1)	3 (1)	1 (1)	
Asian	4 (1)	3 (1)	1 (1)	
Black	40 (13)	32 (14)	8 (10)	
White	121 (39)	83 (37)	38 (48)	
Unknown/Not reported	21 (7)	18 (8)	3 (4)	
**Age, years [median (IQR)]**	**6.0 (2.4–10.2)**	**5.7 (1.8–9.8)**	**6.7 (4.1–11.7)**	**0.02** ∧
Age Category				
** 0–23 months**	**67 (22)**	**59 (26)**	**8 (10)**	**< 0.001** ++
2–4 years old	56 (18)	39 (17)	17 (21)	
5–9 years old	104 (34)	74 (33)	30 (38)	
10–17 years old	73 (24)	51 (22)	22 (28)	
>18 years old	7 (2)	4 (2)	3 (4)	
**UNDERLYING MEDICAL CONDITIONS** *				
Any	203 (66)	146 (64)	57 (71)	0.28
Asthma	80 (26)+	63 (28)	17 (21)	0.16
Chronic lung disease	53 (17)	34 (15)	19 (24)	0.11
Cardiovascular disease	26 (8)	17 (7)	9 (11)	0.36
Renal disease	10 (3)	7 (3)	3 (4)	1
GI/liver disease	7 (2)	6 (3)	1 (1)	0.68
Oncologic	19 (6)	15 (7)	4 (5)	0.6
Hematologic disorder	9 (3)	8 (4)	1 (1)	0.45
**Neurologic disorder (any)**	**74 (24)**	**44 (19)**	**30 (38)**	**0.002**
** seizure disorder**	**37 (12)**	**20 (9)**	**17 (21)**	**0.01**
cerebral palsy	25 (8)	15 (7)	10 (13)	0.15
** cognitive/developmental impairment**	**57 (19)**	**33 (15)**	**24 (30)**	**0.01**
other neurologic disease	37 (12)	24 (11)	13 (16)	0.23
Immunocompromised	22 (7)	20 (9)	2 (3)	0.08
Prematurity	10 (3)	10 (4)	0 (0)	0.6
Obesity (BMI>95% for age)**	41/217 (19)+	31/152 (20)	10/65 (15)	0.45
Other	7 (2)	4 (2)	3 (4)	0.38

# Fisher's exact test.

∧ Wilcoxon sum rank test.

*children may have more than one underlying condition.

**BMI calculated only for children >2 years of age.

+ These conditions were significantly associated (p<0.05) with admission when compared with prevalence rates in Colorado (chi square statistic). Prevalence of asthma and obesity in Colorado in 2008 were reported to be 10% and 13.6%, respectively.

++ Chi square statistic for comparison of children less than 2 years of age to those 2 years of age and older.

Two hundred and three (65%) children had an underlying medical condition, of which asthma (26%), neurological disorders (24%), and obesity (19%) were the most common. Thirty (38%) children hospitalized in the ICU had a neurological condition. In contrast, 44 (20%) children admitted to the floor had an underlying neurological condition (p = 0.002).

### 2009 H1N1 Clinical Features and Hospital Course

The presenting signs and symptoms and laboratory values of hospitalized children with 2009 H1N1 are presented in Table 2. Seizures (p<0.001), mental status changes (p<0.001), decreased breath sounds (p = 0.006), and hypoxia (p = 0.007) were significantly associated with admission to the ICU. Two hundred eighty (87%) children received oseltamivir, with no difference in rates of administration between the ICU and the floor. After the Food and Drug Administration (FDA) issued an Emergency Use Authorization (EUA) for peramivir to treat 2009 H1N1 influenza virus on October 23, 2009, four patients in the ICU received peramivir.

**Table 2:** Clinical characteristics, admission labs, and interventions rendered for hospitalized children with 2009 H1N1 influenza infection.

	TOTAL	FLOOR	PICU	p value*
	N = 307	N = 227	N = 80	
CLINICAL SYMPTOMS	Number (%)	Number (%)	Number (%)	
Day of illness admitted [median (IQR)]	3 (1–5)	3 (2–5)	3 (1–5)	0.46**
Day of fever admitted [median (IQR)]	2 (1–3)	2 (1–4)	1 (0–3)	0.2**
Fever	261 (85)	195 (86)	66 (83)	0.46
Duration [median (IQR)]	3 (2–6)	3 (2–6)	4 (2–6)	0.14**
Vomiting	107 (35)	78 (34)	29 (36)	0.79
Diarrhea	37 (12)	28 (12)	9 (11)	0.85
Shortness of breath/Increased work of breathing	172 (56)	120 (53)	52 (65)	0.07
**Cough**	**254 (83)**	**194 (85)**	**60 (75)**	**0.02**
**Seizures**	**19 (6)**	**7 (3)**	**12 (15)**	**<0.001**
**Mental status changes**	**21 (7)**	**5 (2)**	**16 (20)**	**<0.001**
**PHYSICAL EXAM FINDINGS**				
Crackles	78 (25)	58 (26)	20 (25)	1
Wheezing	74 (24)	53 (23)	21 (26)	0.65
**Hypoxia**	**193 (63)**	**132 (58)**	**61 (76)**	**0.007**
**Duration [median (IQR)]**	**3 (2–5)**	**2 (1–3)**	**6 (3–14)**	**<0.001** **
Tachypnea	164 (53)	115 (51)	49 (61)	0.15
**Decreased breath sounds**	**105 (34)**	**67 (30)**	**38 (48)**	**0.006**
**ADMISSION LABS**				
**WBC ≤4 (10^3^/uL)**	**37/177 (21)**	**28/107 (26)**	**9/70 (13)**	**0.04**
WBC ≥12 (10^3^/uL)	31/177 (18)	16/107 (15)	15/70 (21)	0.31
plat**e**let ≤150 (10^3^/uL)	43/175 (25)	23/107 (21)	20/70 (29)	0.37
platelet ≥500 (10^3^/uL)	4/175 (2)	2/105 (2)	2/70 (3)	1
Creatinine (mg/dL)	8/135 (6)	2/70 (3)	6/65 (9)	0.15
AST ≥180 (U/L)	8/71 (11)	3/37 (8)	5/34 (15)	0.47
ALT ≥120 (U/L)	8/72 (11)	2/37 (5)	6/35 (17)	0.15
elevated LFTs (AST or ALT ≥3× normal)	9/72 (13)	3/37 (8)	6/35 (17)	0.3
bilirubin ≥0.9 (mg/dL)	4/38 (6)	3/35 (9)	1/33 (3)	0.61
**albumin <3.1 (g/dL)**	**21/74 (28)**	**6/37 (16)**	**15/37 (41)**	**0.04**
**CRP ≥1 (mg/dL)**	**52/76 (68)**	**24/42 (57)**	**28/34 (82)**	**0.03**
CRP >10 (mg/dL)	17/76 (22)	7/42 (17)	10/34 (29)	0.27
**median CRP (IQR) (mg/dL)**	**2.2**	**1.3**	**4.1**	**0.004**
**acidotic (blood pH <7.35)**	**51/78 (65)**	**4/15 (27)**	**47/63 (75)**	**0.002**
**MEDICATIONS**				
Oseltamivir	269 (88)	195 (86)	74 (93)	0.11
** 10 day course or longer**	**29 (9)**	**3 (1)**	**26 (33)**	**<0.001**
Time from start of fever to oseltamivir [median (IQR)]	2 (1–4)	2 (1–4)	1 (0–3)	0.11**
Time from admission to oseltamivir [median (IQR)]	0 (0–1)	0 (0–1)	0 (0–1)	0.37**
**Antibioitics**	**201 (65)**	**136 (60)**	**65 (81)**	**<0.001**
Steroids	110 (36)	74 (33)	36 (45)	0.06
Bronchodilators	136 (44)	95 (42)	41 (51)	0.15
**Length of stay [median (IQR)]**	**3 (2–6)**	**2 (2–4)**	**6 (3–17)**	**<0.001** **

*Fisher's exact test.

**Wilcoxon sum rank test.

Thirty-eight (15%) of 247 patients tested by respiratory viral PCR had another virus identified including entero/rhinovirus (31), adenovirus (2), parainfluenza 4 (1), coronaviruses OC-43 (2), and entero/rhinovirus plus parainfluenza (2). Twenty-one patients had a positive blood culture, 8 of which were considered contaminants. Organisms considered true bacteremia included: *Streptococcus pneumoniae* (3), methicillin-susceptible *Staphylococccus aureus (MSSA)* (2), group A *Streptococcus* (1), *Bacillus cereus* (1), *E. coli* (1), *Candida* species (2), *Moraxella catarhalis* (1) *Staphylococcus epidermidis*, (1) and *Streptococcus viridans* (1). Of those with *Streptococcus pneumoniae* bacteremia, one had a lobar pneumonia and one died of meningitis shortly after admission. One patient with MSSA and the patient with GAS bacteremia had positive pleural cultures with these organisms also. *Aspergillus flavus* grew from a post mortem lung culture from one patient who died of respiratory failure.

Multivaratiate logistic regression analysis identified four statistically significant risk factors in hospitalized children associated with admission to the ICU. These included older age (OR 1.07, 1.01–1.13, p = 0.02), underlying neurological condition (OR 2.16, 1.16–4.01, p = 0.02), hypoxia on admission (OR 2.9, 1.15–5.58, p = 0.002), and presenting with mental status changes (OR 12.54, 4.08–38.56, p<0.001).

### Intensive Care Unit Course for 2009 H1N1

The clinical course of children with 2009 H1N1 influenza admitted to the ICU are shown in Table 3. Reasons for admission to the ICU included respiratory distress (81%), seizures and mental status changes (19%), hypotension (19%), sepsis (4%) and other (14%). Most patients admitted to the ICU required ventillatory support, for the others, ICU services were required for either hypotension or management of encephalopathy. Twenty four (30%) of the ICU patients required hemodynamic support. Non invasive ventilation was used in 26 patients (33%) and 32 (40%) children were intubated with a median duration of 9 days. High frequency oscillation was used in 10 patients (13%). Seventeen (53%) of the 32 intubated patients developed acute respiratory distress syndrome (ARDS). Extracorporeal membrane oxygenation (ECMO) was used for 4 patients, with a median duration of 9 days. Eight patients died during their hospital course and one died in the emergency room prior to admission to the hospital (Table 4). The most common cause of death was respiratory failure. Two of the children who died had no underlying medical conditions and only one child who died was less than 2 years of age.

**Table 3:** Comparison of selective characteristics of hospitalized children with 2009 pandemic H1N1 influenza A versus 2009–10 seasonal influenza.

	2009 H1N1 Pandemic (swine) Influenza A	2008–09 Seasonal Influenza A or B	p value*
	N = 307	N = 89	
Male Sex	186 (61%)	47 (53%)	0.22
**Median Age, years [median (IQR)]**	**6.0 (2.4–10.2)**	**1.8 (0.7–8.5)**	**<0.0001** #
**Length of Stay, days [median (IQR)]**	**3 (2–6)**	**4 (2–8)**	**0.02** #
Fever	261 (85%)	74 (83%)	0.74
Hypoxia	193 (63%)	59 (67%)	0.62
Vomitting	107 (35%)	25 (28%)	0.25
**Diarrhea**	**37 (12%)**	**4 (4%)**	**0.047**
Seizures	19 (6%)	8 (9%)	0.47
Mental Status Changes	21 (7%)	10 (11%)	0.18
Any Underlying Medical Condition	203 (66%)	46 (52%)	0.64
**Asthma**	**80 (26%)**	**12 (13%)**	**0.02**
Chronic lung disease	53 (17%)	16 (18%)	0.49
Any neurological condition	74 (24%)	25 (28%)	0.49
seizure disorder	37 (12%)	9 (10%)	0.71
cerebral palsy	25 (8%)	7 (8%)	1
cognitive/development impairment	57 (19%)	21 (24%)	0.36
other neurological condition	37 (12%)	17 (19%)	0.11
CRP [median (IQR)]	2.2	2.8 (0.6–6.6)	0.92#
**Received Antiviral Therapy**	**269 (88%)**	**28 (31%)**	**<0.0001**
Admit to ICU	80 (26%)	27 (30%)	0.5
**Characteristic of Patients Admitted to the ICU**			
	**N = 80**	**N = 27**	
Intubated	32 (40%)	14 (52%)	0.4
High frequency oscillator	10 (13%)	1 (4%)	0.28
**ARDS**	**17 (21%)**	**1 (4%)**	**0.04**
ECMO	4 (5%)	1 (4%)	1
New Disability	14 (18%)	5 (19%)	1
Death	9 (11%)	2 (7%)	0.73

*Fisher's exact test.

# Wilcoxon rank sum test.

**Table 4:** Summary of patients who died secondary to 2009 pandemic influenza A (H1N1) infection.

Sex	Age	Underlying medical condition	Cause of death	Time to death from start of illness	Time of death from admission to the hospital	Received Oseltamivir
M	18 yrs	Dravet's syndrome, developmental delay	Respiratory failure	4 days	2 days	Yes
M	12 yrs	Chromosome 11 mutation Tourette syndrome, asthma, esophageal strictures	Esophageal anastamosis leak, septic shock, ARDS, acute renal failure	5 days	64 days*	No
F	11 yrs	Hypothalamic hamartoma, hydrocephalus, seizure disorder, precocious puberty, diabetes insipidus	Respiratory failure	17 days	12 days	Yes
M	8 yrs	Craniopharyngioma, spastic quadriplegia, asthma	Respiratory failure	5 days	3 days	Yes
M	4 yrs	Seizure disorder	Respiratory failure	30 days	28 days	Yes
F	2 yrs	None	Cardiovascular failure	3 day	1 days	Yes
M	9 months	Hypotonia and developmental delay	Cerebral edema secondary to pneumococcal meningitis	12 days	3 days	Yes
F	12 yrs	New diagnosis of T cell leukemia with this infection	Septic shock/respiratory failure with *Aspergillus flavus* lung infection	27 days	20 days	Yes
F	7 yrs	None	Cardiopulmonary arrest	2 days	Died in ER	No

*infection nosocomially acquired.

### Comparison of 2009 H1N1 Influenza to 2008–09 Seasonal Influenza

The clinical characteristics of hospitalized children with 2009 H1N1 compared to 2008–09 seasonal influenza are shown in Table 3. There were a total of 90 patients admitted with 2008–09 seasonal influenza, 27 (30%) of which were admitted to the ICU. Hospitalized children with 2009 H1N1 influenza were significantly older, had a higher incidence of asthma, were more likely to receive antiviral therapy, had a shorter median length of stay, and had a higher incidence of ARDS compared to children hospitalized with 2008–09 influenza (Table 3).

## Discussion

We report the largest, single-center study of consecutively hospitalized children with 2009 pandemic influenza A (H1N1) virus infection in a tertiary care children's hospital. Children less than 2 years of age have been reported to be at highest risk for hospitalization with seasonal and 2009 H1N1 influenza infections[7], [8], [9], and it has been assumed they would also be at highest risk for admission to an intensive care unit. In support of this, a recent pediatric study from Argentina, reported that children less than 4 years were at highest risk for severe disease with 2009 H1N1 influenza infection [7]. In contrast to these reports, however, our study is the first to demonstrate that older hospitalized children with H1N1 were significantly more likely to require intensive care services than hospitalized children less than 2 years of age. The age group 4–5 years formed the largest group with severe 2009 H1N1 infection requiring intensive care services and the median age of hospitalized children was 6 years. This is in contrast to the 2008–2009 seasonal influenza season, where the median patient age was 1.8 years, and the median age of patients admitted to the PICU was 2.2 years.

The reasons for the peak age differences in disease severity for 2009 H1N1 are unclear. One possible explanation is that younger children may have a less robust immune response to 2009 H1N1 and, therefore, not develop severe pulmonary disease which may be immune mediated. This would imply that the 2009 H1N1 virus elicits a different or more robust immune response than seasonal influenza. It is possible that the youngest children were thought to be at highest risk for severe disease and were targeted for earlier and more aggressive treatment which may have altered the course of their illness. Our study, however, does not support this hypothesis as almost 85% of children, both less than 2 years and greater than 2 years age, received oseltamivir within 24 hours of admission and there were no differences from time of onset of illness to admission between these two groups. Alternatively, in our community, the epidemic peak occurred approximately 6 weeks after the annual reopening of schools (start date for Denver public schools was August 19) which could have resulted in a larger number of older, school-aged children being exposed resulting in a larger number being infected with 2009 H1N1, some of whom developed severe infection.

Our study found that having an underlying neurologic disorder was a risk factor for admission to the ICU with 2009 H1N1. Interestingly, half of the patients in the intensive care unit who had underlying neurologic disorders did not have any associated neuromuscular or pulmonary disorders such as asthma, chronic aspiration, or recurrent pneumonia. These findings support and strengthen the recommendations that this group of patients with 2009 H1N1 infection need to be aggressively targeted for immunization, prophylaxis, early recognition, and prompt treatment.

Viral co-infections and secondary bacterial infections were infrequent in our H1N1 cohort. The low incidence of other respiratory viruses may be explained by the fact that our peak of illness occurred in the early fall before our winter respiratory viral season. Although the majority of children with H1N1 admitted to the ICU were started on broad spectrum antibiotics, very few presented with or developed any clinical or laboratory evidence of secondary bacterial infections.

In our study, 26% of the hospitalized children with 2009 H1N1 were admitted to the ICU. Although our ICU admission rates were not different between 2009 H1N1 and 2008–09 seasonal influenza, it is in contrast to the 2003–04 influenza season, the most recent severe influenza epidemic in the United States, where a multi-state study reported 11% of hospitalizations in children resulted in ICU admission [10]. The increased use of ICU services associated with children with 2009 H1N1 infection was also supported by a recent study in Atlanta which showed an increase in the proportion of ICU admission for children presenting to the emergency department with influenza-like illness compared with two previous influenza seasons [11].

More than half of the intubated children with H1N1 in our study developed ARDS compared to 7% of seasonal influenza patients. Additionally, there was a trend toward higher use of high frequency ventilation compared to the previous season. This is in contrast to the pediatric study from Argentina where only 17% of the ICU admissions required mechanical ventilation[7], but similar to a children's case series from Johns Hopkins in which 46% required mechanical ventilation [12]. We speculate that the high incidence of ARDS in school age children may be related to an excessive immune response mediated by cytokines to this novel strain of influenza. Further studies are needed to determine whether particular virulence factors unique to the 2009 pandemic influenza A (H1N1) virus strain or host response to the virus explains this high severity of pulmonary dysfunction.

Interestingly, our study showed a longer hospitalization among children admitted with 2008–09 seasonal influenza compared to children hospitalized with 2009 H1N1. This may be explained by a significantly lower rate of receipt of antiviral therapy in hospitalized children with seasonal influenza compared with 2009 H1N1 influenza. For 2009 H1N1, the hospital adopted a recommendation to initiate antiviral therapy on any hospitalized patient with influenza, compared to the prior influenza season, when patients were only started on antiviral medication if they presented in the first 48 hours of symptoms or if they were severely ill.

There are several limitations of our study. Our hospital is the only academic tertiary children's hospital in the state of Colorado and serves as a referral center for neighboring states, which might explain our higher admissions to the ICU. Second, for 2009 H1N1 there were fifty-one patients included in our study that had a positive influenza IA, but did not have enough sample left for confirmatory PCR. Therefore, we may have included a very small number of patients who had a falsely positive influenza IA test. Based on our laboratory's experience with other specimens, however, we estimate that at most we may have had 9 false positives, 3% of our total study population. Additionally, we performed a subanalysis on the patients who were only PCR positive, which did not alter any of our findings. Third, although PCR testing was implemented during the 2008–09 season, a lower percentage of samples had confirmatory PCR testing performed. Finally, this study looked only at hospitalized children and therefore does not reflect the full spectrum of disease caused by H1N1 as seen in the community.

In summary, in 2009 H1N1 hospitalized children, older age and having an underlying neurological medical condition were significant independent risk factors for development of severe disease. In general, hospitalized children with H1N1 who were admitted to the ICU had a severe disease course, as evidenced by the high use of oscillatory ventilation and a high incidence of ARDS. The comparison to seasonal influenza highlights the difference in age groups hospitalized with H1N1 and the increased severity of pulmonary disease. These observations identify new high risk groups of children who might benefit from increased emphasis on prevention, earlier recognition, and new treatment strategies for H1N1 infections.
